# Spectral photoacoustic imaging to estimate *in vivo* placental oxygenation during preeclampsia

**DOI:** 10.1038/s41598-018-37310-2

**Published:** 2019-01-24

**Authors:** Dylan J. Lawrence, Megan E. Escott, Leann Myers, Suttira Intapad, Sarah H. Lindsey, Carolyn L. Bayer

**Affiliations:** 1 Department of Biomedical Engineering, Tulane University, 500 Lindy Boggs Center, New Orleans, LA 70118 USA; 2 School of Public Health and Tropical Medicine, Tulane University, 1440 Canal St #2400, New Orleans, LA 70112 USA; 3 School of Medicine, Tulane University, 1430 Tulane Ave, New Orleans, LA 70112 USA

## Abstract

Preeclampsia is a pregnancy-related hypertensive disorder accounting for 14% of global maternal deaths annually. Preeclampsia — maternal hypertension and proteinuria — is promoted by placental ischemia resulting from reduced uteroplacental perfusion. Here, we assess longitudinal changes in placental oxygenation during preeclampsia using spectral photoacoustic imaging. Spectral photoacoustic images were acquired of the placenta of normal pregnant (NP) and preeclamptic reduced uterine perfusion pressure (RUPP) Sprague Dawley rats on gestational days (GD) 14, 16, and 18, corresponding to mid- to late gestation (n = 10 per cohort). Two days after implementation of the RUPP surgical model, placental oxygen saturation decreased 12% in comparison with NP. Proteinuria was determined from a 24-hour urine collection prior to imaging on GD18. Blood pressure measurements were obtained on GD18 after imaging. Placental hypoxia in the RUPP was confirmed with histological staining for hypoxia-inducible factor (HIF)-1α, a cellular transcription regulator which responds to local oxygen levels. Using *in vivo*, longitudinal imaging methods we determined that the placenta in the reduced uterine perfusion pressure rat model of preeclampsia is hypoxic, and that this hypoxia is maintained through late gestation. Future work will utilize these methods to assess the impact of novel therapeutics on placental ischemia and the progression of preeclampsia.

## Introduction

Pregnancy-related hypertensive disorders, including preeclampsia, account for 14% of global maternal deaths annually^1^. Preeclampsia is clinically diagnosed as pregnancy related high blood pressure of more than 140/90 with proteinuria greater than 300 mg in 24 hours. It is believed that an initiating condition of the disease is placental ischemia resulting from reduced uteroplacental perfusion, potentially due to abnormal placental development. During the early stages of placental development, if the uterine vasculature fails to properly remodel and the invasion of the embryonic trophoblasts into the maternal endometrium is insufficient, the resulting placenta may not provide sufficient maternal-fetal exchange, leading to placental ischemia. Placental ischemia, in turn, increases expression of hypoxia inducible factors (HIFs) and increases production of soluble antiangiogenic factors which induce maternal endothelial dysfunction by inhibiting vasorelaxation signaling, leading to maternal hypertension^2,3^. Placental ischemia is therefore a critical indicator in the progression of preeclampsia. Clinically, placental ischemia can be assessed by measuring blood oxygen levels through cordocentesis. However, this highly invasive procedure poses risks to fetal well-being. Noninvasive imaging offers an alternative approach for the detection of placental ischemia, indicative of preeclamptic risk, prior to the onset of maternal symptoms.

Photoacoustic imaging is a hybrid light and sound imaging modality capable of providing high spatial and temporal resolution images. In recent years, it has been utilized for a range of biomedical imaging applications including ophthalmology and oncology^4,5^. Photoacoustic imaging uses nanosecond pulsed laser light in the near infrared to capitalize on the increased imaging depth available in this tissue optical window. The light is absorbed by chromophores in the tissue, such as endogenous hemoglobin, generating photoacoustic transients which are then received by an ultrasound transducer.

Spectral photoacoustic imaging can distinguish between oxygenated and deoxygenated perfused tissues by estimating the wavelength-dependent absorption of the tissue. Using a linear least-squares spectral unmixing algorithm, the resulting photoacoustic signal intensity can be fit to characterize the absorption of oxyhemoglobin (Hb) and deoxyhemoglobin (HB_O2_). From this information, the relative oxygen saturation in the imaged area can be calculated. This technique has primarily been utilized to measure hypoxia in applications in oncology^6^. Recently, there has been an increased interest in adapting spectral photoacoustic imaging methods for the preclinical measurement of placental oxygenation. Spectral photoacoustic imaging has been used to detect placental and fetal oxygen saturation in normal and pathologic pregnancies and to investigate the effect maternal hypoxia and hyperoxygenation have on placental function^7,8^. This work has demonstrated spectral photoacoustic imaging as a powerful tool to expand the preclinical understanding of pregnancy-related diseases.

The reduced uterine perfusion pressure (RUPP) model is a well-established model of preeclampsia that induces placental ischemia through surgical clipping of the abdominal aorta and uterine arteries^9^. The mechanical reduction in uteroplacental blood flow causes vascular dysfunction, leading to placental hypoxia. The mechanism of disease development in the RUPP model replicates many aspects of the placental-driven onset of the human condition^9,10^. RUPP animals show the clinical symptoms of preeclampsia during late gestation along with increased levels of hypoxia inducible factor (HIF)-1α, a cellular transcription regulator which responds to local oxygen levels to control vascularization and angiogenesis^2^. Here, we demonstrate that spectral photoacoustic imaging can be used to measure placental ischemia in the RUPP model of preeclampsia.

## Materials and Methods

### Animal Studies

All animal studies were conducted following protocols approved by the Institutional Animal Care and Use Committee at Tulane University. Timed pregnant Sprague Dawley rats were acquired from a commercial vendor (Charles River Laboratories, Boston, MA) and randomly assigned to experimental groups. Animals undergoing the RUPP procedure (n = 10) were anesthetized on gestational day (GD) 14 under 2% isoflurane. An abdominal midline incision was made and both uterine horns were exteriorized. A silver clip (0.203 mm ID) was placed around the abdominal aorta between the renal arteries and iliac bifurcation. To prevent a potential adaptive increase in uterine blood flow, secondary clips (0.100 mm ID) were placed on both ovarian arteries proximal to the branch to the first pup resulting in an approximated 40% reduction in uteroplacental perfusion^9^. A successful procedure was defined as a minimum of one viable pup at the time of euthanization on GD18. A sham surgical group (n = 10) underwent a similar procedure but no clips were placed. A daily 1-mg/kg subcutaneous injection of carprofen was administered for postoperative pain management.

### Photoacoustic and Ultrasound Imaging

**Figure 1:**
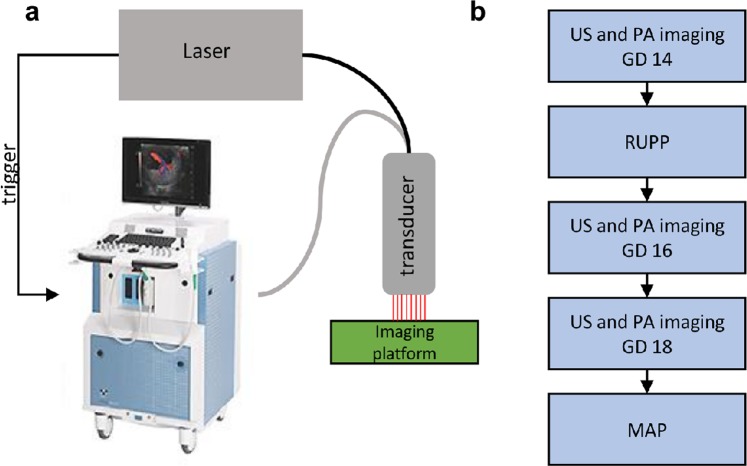
(**a**) The custom-integrated US and photoacoustic imaging system (image of Vevo 2100 reproduced with permission from FUJIFILM VisualSonics). The Phocus Benchtop laser triggers the dual acquisition of US and PA images. Images are acquired using an ultrasound transducer integrated with a fiberoptic bundle for laser light delivery. (**b**) describes the sequence of the experimental procedures.

Imaging was performed using a Vevo 2100 ultrasound system (FUJIFILM VisualSonics, Toronto, Ontario, Canada) custom-integrated with an Opotek Phocus HE Benchtop laser (Opotek, Carlsbad, CA) shown in Fig. 1. Beginning on GD 14, animals were anesthetized with isoflurane, abdominal hair was removed with depilatory cream, and animals were transferred to a heated physiologic imaging platform (FUJIFILM VisualSonics). Heart rate, respiration rate, and body temperature were monitored and maintained throughout imaging. An LZ-250 linear array transducer (256 elements, 13–24 MHz broadband frequency, 20 MHz center frequency) was used to acquire all ultrasound (US) and photoacoustic images in a 30 × 22 mm area. An integrated fiberoptic bundle was used to deliver between 10 and 20 mJ/cm^2^ of light over the wavelengths imaged. A very small piece of aluminum foil was placed between the ultrasound transducer and the gel coupling the transducer to the skin; the reflective foil acts to reduce artifacts in the photoacoustic image originating from light reflections from the skin^11^. Color Doppler ultrasound was used to find the location of the umbilical cord insertion, to identify the midline of the placenta, which defined the imaging plane.

Five placentas per animal were imaged on GD 14 (prior to surgical intervention), 16, and 18. Placentas were located using B-mode ultrasound by scanning each side of the abdomen from bladder to ribcage. Placentas were selected for PA imaging if a complete placental border was visible in the US image; in a typical imaging session, a small portion of the placentas would be blocked by artifacts from maternal intestines and would be excluded from the study. In animals with five or fewer viable pups, all placentas meeting the criteria were imaged. If more than five pups were viable, placentas distributed across the entire abdomen were selected for imaging to account for possible regional differences in placental perfusion. Following the RUPP procedure, placentas located under the sutures were also excluded from imaging due to the photoacoustic signal interference generated by the suture line. Photoacoustic images with co-registered B-mode ultrasound images of anatomy were acquired at 690, 808, and 950 nm, corresponding to the relative optical absorption peaks of deoxyhemoglobin, the isosbestic point, and oxyhemoglobin respectively. Animals were allowed to recover from anesthesia for two days between imaging sessions.

### Spectroscopic Processing

All US and photoacoustic images were exported to Matlab (Mathworks, Natick, MA) and parsed into data matrices for processing and display. B-mode US images were used to manually define placental tissue. A linear least-squares spectral unmixing algorithm was performed within this region of interest to estimate the concentration of Hb and Hb_O2_^6,12^. Oxygen saturation was calculated using the average concentrations found within the region of interest:

1 $$s{O}_{2}=\frac{C[H{b}_{O2}]}{C[H{b}_{O2}]+C[Hb]}$$

A custom 255-value oxygenation colormap — red representing completely oxygenated blood and blue completely deoxygenated blood— was then superimposed on the B-mode US image to display the calculated sO_2_ in the region of interest. The transparency of the oxygenation image was scaled to the laser fluence-corrected photoacoustic signal.

### Blood Pressure Measurements

On GD 18, animals (n = 6) were anesthetized under isoflurane and a saline-filled catheter was inserted in the left common carotid artery with the tip located near the left ventricle of the heart. The catheter was then connected to a pressure transducer and a computer data acquisition interface (PowerLab, ADInstruments, Colorado Springs, CO). Blood pressure recordings were performed for 10 minutes while the animal remained under anesthesia. To reduce potential variation in blood pressure due to length of exposure to isoflurane^13^, all MAP measurements began 35 minutes after induction of anesthesia. MAP was also measured in a pilot group of animals (n=3) on GD16 and 18 after insertion of a carotid catheter at GD14 during the RUPP surgery; due to complications of maintaining the carotid artery catheter patency for an extended period of time, the carotid catheter placement was moved to GD18 for the full study.

### Protein Analysis

During the 24-hour period prior to the blood pressure measurements, animals were housed in hydrophobic sand bedding (labsand; Coastline Global Inc., Palo Alto, CA). All urine was collected at the end of the period and stored at −20 °C until processed. A Micro-Lowry total protein kit (Sigma-Aldrich, St. Louis, MS) was used to determine protein excreted in the urine. Samples were thawed and gently agitated to homogenize the sample. A 200 µL sample was taken and diluted to 1 mL in a microcentrifuge tube. Deoxycholate was added to each sample followed by trichloroacetic acid and samples were then centrifuged, decanted, and supernatants removed. The pellets were dissolved in 1 mL of Lowry reagent solution. The microcentrifuge tubes were rinsed with 1 mL of water that was then returned to its respective sample. After allowing the samples to rest, 0.5 mL of Folin & Ciocalteu’s Phenol Reagent Solution was added to each sample and color was developed for 30 minutes. Optical absorbance was read on a SpectraMax i3x microplate reader (Molecular Devices, Sunnyvale, CA). Protein concentration was calculated for each sample from a standard curve generated at 500 nm and multiplied by the volume of urine collected to determine the amount of protein excreted in the 24-hour period.

### Immunohistochemistry

Following the acquisition of images on GD 18, animals were euthanized with an overdose of CO_2_. One placenta per animal was collected and frozen in a cryo-embedding solution prior to sectioning into 4 µm slices. Immunohistochemical staining was performed on whole placental sections using the Avidin Biotin Complex (ABC) method^14^ for mouse monoclonal HIF-1α (abcam, Cambridge MA). Briefly, slides were bathed in xylene followed by descending concentrations of ethanol, deionized water, a 0.3% hydrogen peroxide solution, phosphate buffered saline (PBS), and a blocking serum. The anti-HIF-1α primary antibody was diluted in blocking serum and slides were incubated overnight. The following day slides were rinsed in PBS and incubated with streptavidin/peroxidase followed by 3,3′-diaminobenzidine (DAB). Color was allowed to develop for 15 minutes, slides were rinsed in a running water bath for 10 minutes, and then counter-stained with hematoxylin. Microscopy was performed with an Olympus BX51 (Center Valley, PA) microscope and images were exported to ImageJ (National Institutes of Health, Bethesda, MD) for processing. The placental border of the stained whole placental sections was segmented manually from the microscopy images, and a color deconvolution was applied to separate the DAB and hematoxylin signals. HIF-1α expression was quantified as the percent area fraction of DAB staining to the total area of the placental section.

### Statistical Analysis

Sample sizes were determined from an a priori power analysis in G*Power Software (Heinrich-Heine-Universität Düsseldorf, Düsseldorf, Germany). A sample size of 10 achieved an actual power of 0.958 with an effect size of 1.569. For each gestational day the five placental sO_2_ measurements were averaged. A two-way repeated measures ANOVA was performed in R software (R Foundation for Statistical Computing, Vienna, Austria). A *P* value of 0.05 was used to determined statistical significance. Measured sO_2_ values are reported as mean ± standard error (SEM).

A pairwise t-test with a Bonferroni correction was also conducted on each group to investigate the significance (α = 0.05) of relative changes in placental sO_2_ across gestational day. The datasets generated during and/or analyzed during the current study are available from the corresponding author on request.

## Results

### Photoacoustic and US Imaging

**Figure 2:**
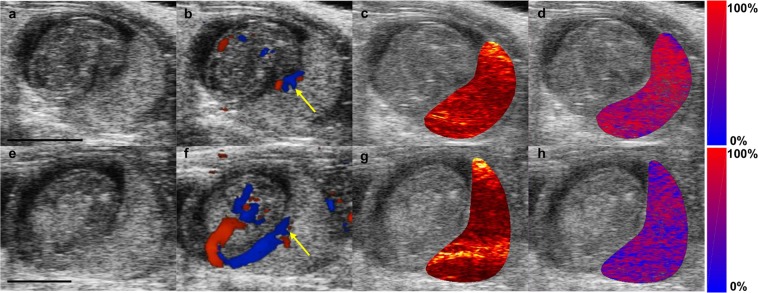
B-mode US images of the placental environment, (**a**,**e**), in NP, (**a**–**d**), and the RUPP, (**e**–**h**), at GD 16. (**b,f**) show the Color Doppler ultrasound of blood flow the umbilical cord. The yellow arrow indicates the origin of the umbilical cord on the placenta which was used as a landmark for determining the imaging field. (**c**,**g**), show the accumulation of the photoacoustic signal intensity at 808 nm segmented to the placental. At the isosbestic point, the photoacoustic signal is generated equally by both Hb and Hb_O2_. The custom colormap of oxygen saturation, segmented to the placenta and superimposed on the ultrasound image, is shown in (**d**,**h**). Red denotes completely oxygenated blood while blue denotes completely deoxygenated blood. Scale bars are 3 mm.

Figure 2 shows representative images of RUPP and NP placental environments on gestational day 16. Co-registered ultrasound provides high-resolution images of the developing anatomy (Fig. 2a,e). Color Doppler ultrasound (Fig. 2b,f) shows blood flow in the umbilical cord used as an identifier of the placental midline for selecting the imaging area. An overlay of the photoacoustic signal at 808 nm, the isosbestic point of hemoglobin, on the US image (Fig. 2c,g) shows the anatomical origin of photoacoustic transients in the placenta. Spectral photoacoustic imaging of the same anatomical environment and a linear least-squares spectral unmixing algorithm characterized the concentration of Hb and Hb_O2_ in the placenta. An oxygenation colormap, segmented to the placenta and superimposed on the ultrasound images (Fig. 2d,h) shows the RUPP animals have more prominent blue coloring, indicating decreased oxygenation, compared to NP animals.

**Figure 3:**
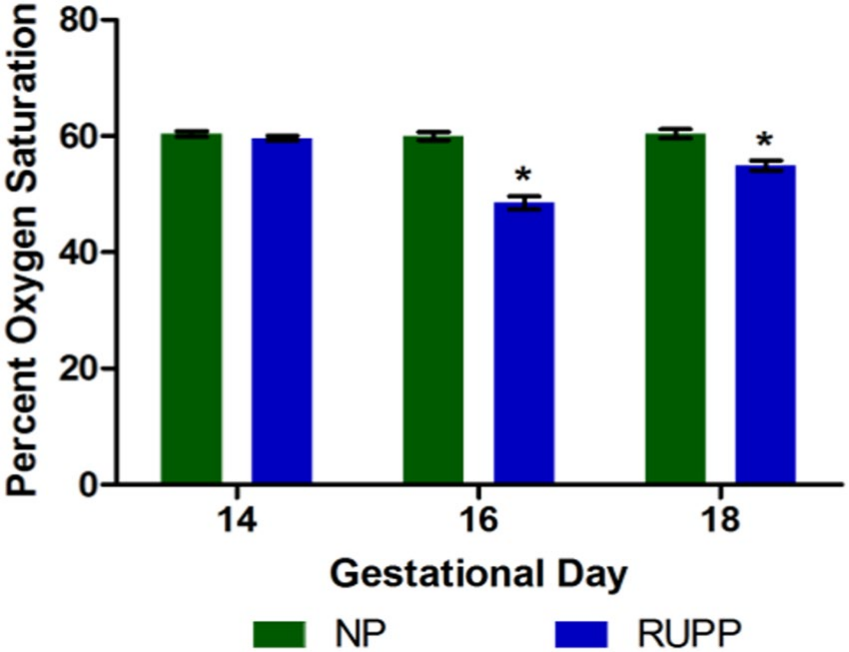
Mean placental oxygen saturation was significantly lower (p < 0.05) in the RUPP two days after surgery compared to NP. This significant difference was maintained through GD 18.

Two days after surgical intervention (GD 16), average placental oxygenation was significantly lower (p < 0.05) in the RUPP compared to normal pregnant animals (Fig. 3). Four days after surgical intervention (GD18), the average placental oxygenation remains significantly lower (p < 0.05) in the RUPP compared to normal pregnant animals; however, a slight recovery is observed in the estimated placental sO_2_ values in the RUPP. Placental compensation through increased vascular growth and remodeling may be responsible for this slight recovery four days after surgery; that the high mean arterial pressure is maintained is indicative that the RUPP placenta is still ischemic in comparison to the normal pregnant placenta.

Mean maternal weight gain from GD 14 to 18 was significantly lower (p < 0.05) in the RUPP than NP rats (Table 1), likely due to reabsorption of a portion of the pups following the surgical modification. Pup and placental weights did not differ between RUPP and normal pregnant liters at the time of dissection, consistent with previously reported data^15^. The Sham surgical group exhibited no significant differences from the NP group (data not shown).

**Table 1:** Maternal and fetal characteristics in RUPP and NP animals. Maternal weight gain was measured as the difference in body weight between GD 14 and GD 18. RUPP animals gained less weight on average likely due to the resorption of a portion of pups due to the surgical procedure.

	Normal Pregnant (n = 10)		RUPP (n = 10)		P value
	Mean	± SEM	Mean	± SEM	
Maternal Weight Gain, g	25.112	±2.92	10.40	±1.09	<0.001*
Pup Weight, g	1.542	±0.01	1.502	±0.08	0.239
Placenta Weight, g	0.486	±0.01	0.469	±0.04	0.432

Pup and placenta weights were measured post mortem on GD 18. While average RUPP pup and placenta weights were lower than NP, no significant differences were found, consistent with previously reported data^15^. P value of less than 0.05 determined statistical significance*. Data shown as mean ± SEM.

### Blood Pressure Measurements

**Figure 4:**
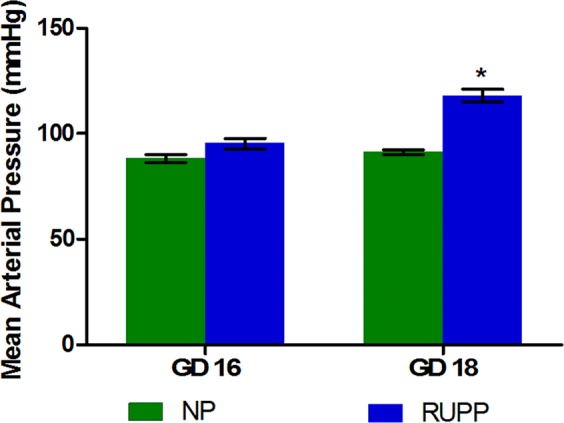
Mean arterial pressure in the RUPP model was significantly higher (p < 0.05) at GD 18 compared to NP. A pilot group of animals (n = 3) showed MAP in the normal range on GD 16. All data is reported as mean ± SEM.

Mean arterial pressure (MAP) was measured through a saline filled catheter placed in the common carotid artery on GD 18 while the animals were anesthetized. Figure 4 illustrates the average MAP in RUPP of 118 ± 3 mmHg was significantly higher (p < 0.05) in comparison with an average of 91 ± 1 mmHg in the normal pregnant group on GD 18. In the pilot study, which placed the catheters at GD 14, the GD 16 RUPP animals MAP averaged 95 ± 2 mmHg, while the normal pregnant GD 16 MAP was 88 ± 2 mmHg.

### Protein Analysis

**Figure 5:**
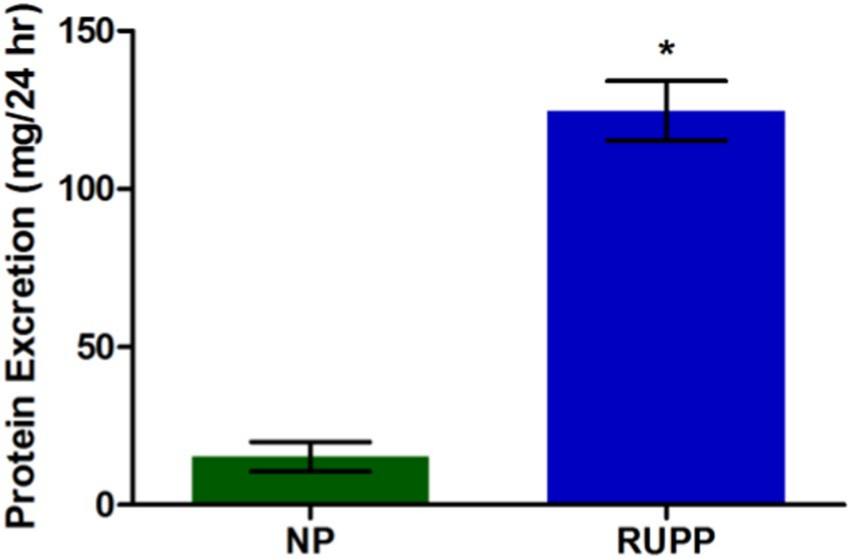
Average protein excretion in the RUPP (124 ± 9.44) was significantly higher (p < 0.05) than NP (15.27 ± 4.66) in urine samples collected during the 24-hour period prior to MAP measurements.

Protein excretion measured in urine samples collected from the 24-hour period prior to MAP measurements are shown in Fig. 5. The RUPP group exhibited significantly elevated (p < 0.05) levels of total protein excreted compared to NP, indicating RUPP animals exhibit proteinuria, a symptom of human preeclampsia. These findings are consistent with those previously reported in literature^10^.

### Immunohistochemistry

**Figure 6:**
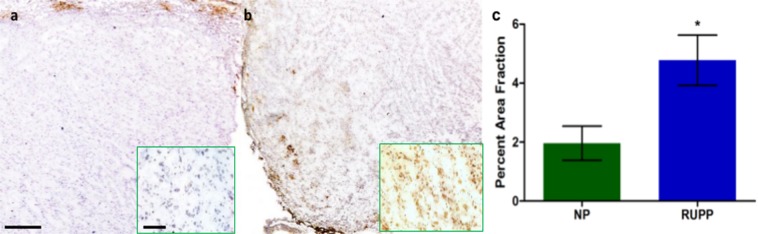
Immunohistochemical staining for HIF-1α counterstained with hematoxylin in the placenta. The expression of HIF-1α in the RUPP (**b**) was significantly higher (p < 0.05) compared to NP (**a**) observed by the increased DAB brown coloring. Insets were acquired with a 40x objective. (**c**) Quantification of the HIF-1α staining. Scale bars = 0.5 mm, and 0.1 mm for insets.

Placental sections were stained for HIF-1α, a cellular protein regulator of oxygen homeostasis (Fig. 6). Elevated levels of HIF-1α are normal in early human development but fall around 10 weeks gestation corresponding to approximately GD 14 in rats. HIF-1α expression remains elevated in preeclampsia, which may contribute to continued shallow trophoblast invasion and reduced placental perfusion^16^. RUPP placentas harvested at GD 18 showed significantly elevated (p < 0.05) levels of HIF-1α compared to NP animals at the same time point.

## Discussion

The aim of this study was to use spectral photoacoustic imaging to monitor placental oxygenation levels in an *in vivo* animal model of preeclampsia. We found that the placental environment was hypoxic two days after the RUPP procedure was implemented and that hypoxia was maintained through GD 18 in comparison with normal pregnant animals. Mean arterial pressure measures on GD 16 suggest RUPP animals are normotensive at the time placental hypoxia occurs and develop hypertension later in gestation. At this time point, the RUPP animals were still receiving post-surgical analgesics which could be responsible for reducing the blood pressure to normal limits. Using noninvasive imaging techniques, we have shown that the hypoxic placental environment during mid gestation resulted in hypertension and proteinuria during late gestation. Attenuation of light through tissue places limitations on the accuracy and sensitivity of this technique to deep tissue structures (greater than 2 to 3 cm below the skin surface). However, the field is progressing rapidly and potential approaches to overcome the imaging depth limitations include using laser in the near infrared-II wavelength range^17^, optimizing light delivery to minimize artifacts and noise^18^, and implementation of machine learning image processing techniques to further improve signal to noise ratios^19^.

A recent clinical trial assessed utility of photoacoustic imaging for diagnostic staging of breast cancer^20^. For placental imaging, early stage anatomy may be more accessible using a transvaginal probe^21^, or an endoscope during fetal surgery^22^. Careful consideration of light delivery would be needed to ensure the safety of the developing fetus. Other noninvasive imaging techniques, such as blood oxygen level-dependent (BOLD) MRI, have been investigated for measuring placental oxygenation in both preclinical and clinical settings^23–26^. BOLD MRI is based on the principle that changes in oxygen saturation lead to changes in local magnetic field susceptibility and therefore detects a relative change in the MRI signal rather than directly measuring hemoglobin oxygen saturation.

The RUPP rat is a robust and well-characterized model for investigating preeclampsia. In addition to hypoxia, RUPP animals also exhibited hypertension, proteinuria, and elevated levels of HIF-1α in the placenta on GD18^8,27^. Other animal models of preeclampsia such as L-N^G^-Nitroarginine methyl ester (L-Name), induce preeclamptic symptoms through chronic nitric oxide synthase inhibition^8^. However, the dose-dependent hypertension produced by the L-Name treated animals does not model the placental signaling believed to initiate the human disease^28^. The mechanical reduction in placental blood flow in the RUPP makes it ideal for validation of our spectral photoacoustic imaging methods to detect placental ischemia.

In the present study we found the normal pregnant rats to have an average placental oxygenation of 60%, similar to previously reported photoacoustic imaging of pregnant mice^8^. Human pregnancy benchmarks often come from pregnant sheep because of commonalities in placental development and metabolic function. Placental and fetal oxygen content, measured via indwelling catheters in maternal and fetal sheep circulation, are comparable to human blood samples obtained from cordocentesis^29,30^. Based on the blood oxygen saturation found in conscious pregnant sheep and assuming an equal distribution of maternal and fetal blood, the placenta has an average oxygen saturation of approximately 70% from mid to late gestation^31^. In the current study and the prior studies in mice, the imaging was performed under isoflurane suggesting that anesthesia could be responsible for the decreased oxygenation measured in rodents compared to the human and sheep studies. While heart rate and respiration rate were kept in the same range for all US and PA image acquisition, it is possible that changes in anesthetic response after the RUPP procedure could influence local oxygen levels in the placenta.

In this work we have used spectral photoacoustic imaging methods to longitudinally monitor placental oxygenation *in vivo* in the RUPP model of preeclampsia. We have demonstrated the role of placental hypoxia in the development of preeclampsia in the RUPP model. Spectral photoacoustic imaging of placenta function could conceivably be used in the future to assess placental ischemia in preclinical models of gestational diabetes, fetal growth restriction, and preeclampsia, informing efficacy of potential therapeutic treatment. Our future work will use spectral photoacoustic imaging to investigate the effect of therapeutic intervention on placental oxygen levels *in vivo*. Spectral photoacoustic imaging is a powerful preclinical tool that has many promising applications in the understanding and treatment of pregnancy related diseases.
